# Aphid-Responsive Defense Networks in Hybrid Switchgrass

**DOI:** 10.3389/fpls.2020.01145

**Published:** 2020-07-30

**Authors:** Kyle G. Koch, Nathan A. Palmer, Teresa Donze-Reiner, Erin D. Scully, Javier Seravalli, Keenan Amundsen, Paul Twigg, Joe Louis, Jeffrey D. Bradshaw, Tiffany Marie Heng-Moss, Gautam Sarath

**Affiliations:** ^1^ Department of Entomology, University of Nebraska at Lincoln, Lincoln, NE, United States; ^2^ Wheat, Sorghum, and Forage Research Unit, USDA-ARS, Lincoln, NE, United States; ^3^ Department of Agronomy and Horticulture, University of Nebraska at Lincoln, Lincoln, NE, United States; ^4^ Biology Department, West Chester University of Pennsylvania, West Chester, PA, United States; ^5^ Stored Product Insect and Engineering Research Unit, USDA-ARS, Manhattan, KS, United States; ^6^ Redox Biology Center, Department of Biochemistry, University of Nebraska at Lincoln, Lincoln, NE, United States; ^7^ Biology Department, University of Nebraska at Kearney, Kearney, NE, United States; ^8^ Department of Biochemistry, University of Nebraska-Lincoln, Lincoln, NE, United States

**Keywords:** *Panicum virgatum*, hybrid switchgrass, aphids, plant defense, transcriptomes, gene-networks, transcription factors, metabolites

## Abstract

Aphid herbivory elicits plant defense-related networks that are influenced by host genetics. Plants of the upland switchgrass (*Panicum* *virgatum*) cultivar Summer can be a suitable host for greenbug aphids (*Schizaphis graminum*; GB), and yellow sugarcane aphids (*Sipha flava*, YSA), whereas the lowland cultivar Kanlow exhibited multi-species resistance that curtails aphid reproduction. However, stabilized hybrids of Summer (♀) x Kanlow (♂) (SxK) with improved agronomics can be damaged by both aphids. Here, hormone and metabolite analyses, coupled with RNA-Seq analysis of plant transcriptomes, were utilized to delineate defense networks induced by aphid feeding in SxK switchgrass and pinpoint plant transcription factors (TFs), such as WRKYs that potentially regulate these responses. Abscisic acid (ABA) levels were significantly higher in GB infested plants at 5 and 10 days after infestation (DAI). ABA levels were highest at 15DAI in YSA infested plants. Jasmonic acid levels were significantly elevated under GB infestation, while salicylic acid levels were signifi40cantly elevated only at 15 DAI in YSA infested plants. Similarly, levels of several metabolites were altered in common or specifically to each aphid. YSA infestation induced a significant enrichment of flavonoids consistent with an upregulation of many genes associated with flavonoid biosynthesis at 15DAI. Gene co-expression modules that responded singly to either aphid or in common to both aphids were differentiated and linked to specific TFs. Together, these data provide important clues into the interplay of metabolism and transcriptional remodeling accompanying defense responses to aphid herbivory in hybrid switchgrass.

## Introduction

Insect herbivores can impose significant costs to plant fitness (Zust and Agrawal, 2017; Nalam et al., 2019). Aphids are especially important plant pests that often have broad host ranges and significantly reduce growth/yields of susceptible plants *via* nutrient depletion and feeding damage to host tissues (Smith and Chuang, 2014). Aphid feeding triggers host defense responses arising from mechanical stimuli, plant tissue damage, salivary secretions, and removal of nutrients (Kindt et al., 2006; Zust and Agrawal, 2016). Plant defensive responses are influenced by the genetics of the host and the nature of the plant-aphid interactions. For example, in aphid-tolerant plants, defensive responses are modulated following initial infestation, allowing recovery of plant growth without a significant impact on aphid reproduction, whereas in susceptible plants, aphid herbivory can lead to significant plant yield/biomass losses or even plant death (Koch et al., 2016). Although much progress has been made in understanding the host defensive-networks underlying responses to aphids in model systems (Nguyen et al., 2016 *;* Zust and Agrawal, 2017), there is still a significant lack of data on insect herbivore interactions with non-model plants and host signaling networks that influence defense responses at later stages of aphid infestation. Additionally, aphids can modulate plant defensive responses by secreting elicitors and proteins that improve their survival (Kaloshian and Walling, 2016).

Several omics strategies alone or in combination have been used to evaluate plant-aphid, and plant-insect interactions [for example: (Wu and Baldwin, 2010; Maag et al., 2015b; Tzin et al., 2015; Zhou et al., 2015; Wozniak et al., 2017; Kiani and Szczepaniec, 2018; Erb and Reymond, 2019; Nalam et al., 2019; Sanchez-Arcos et al., 2019; He et al., 2020; Zhang et al., 2020; Zogli et al., 2020)]. In general, these studies have shown extensive changes in the transcriptomes and metabolomes upon insect herbivory. A common factor in many of these interactions has been the documentation of changes in the levels of plant hormones such as jasmonic acid (JA), its active form JA-isoleucine (JA-Ile), and a precursor of JA, 12-oxo-phytodienoic acid (OPDA), along with other hormones such as salicylic acid (SA), abscisic acid (ABA), and indole acetic acid (IAA). Hormone levels can be differentially changed depending on the pest, the host plant, and timing of analyses. Expression levels of transcription factors (TFs) that are regulated by changes in cellular physiology are subsequently impacted. TF families that are known to have roles in regulating plant defensive networks including WRKYs, MYBs, ERFs, NACs, bHLHs, and bZIPs, which, in turn, regulate expression levels of their cognate genes and pathways. The net result of these changes in TF expression levels is the modulation of primary and secondary metabolic pathways, which serve to trigger defense responses to mitigate herbivory. Although several components of defense responses are conserved among multiple plant species, plant genotype, environment, and other factors can lead to variations in the cellular metabolism of the same hosts challenged by insect herbivores (Coppola et al., 2013; Maag et al., 2015a; Castano-Duque and Luthe, 2018; Muneer et al., 2018; Sanchez-Arcos et al., 2019; Zhao et al., 2019). Despite variations in metabolite profiles, certain pathways and compounds produced by these pathways are known to confer protection to plants against biotic stressors. These include reactive oxygen and nitrogen intermediates, glucosinalates, alkaloids, terpenoids, flavonoids, phenypropanoids, and chemicals derived from amino acids, such as pipecolic acid. Pipecolic acid is both an antifeedant as well as a molecule required for triggering systemic acquired resistance (SAR) (Cecchini et al., 2015; Hartmann and Zeier, 2018; Hartmann and Zeier, 2019). The plethora of documented plant defensive strategies serves as a good platform to evaluate a potential role for similar mechanisms, genes, and metabolites in the defense responses of switchgrass to aphids.

Switchgrass (*Panicum virgatum* L.) is an herbaceous non-model species targeted for bioenergy but with important ecosystem advantages as a conservation and forage crop (Vogel et al., 2011). It also has an annotated genome (https://phytozome.jgi.doe.gov/pz/portal.html#!info?alias=Org_Pvirgatum_er) that has facilitated a better understanding of genomic and functional genomic aspects of this plant [for example: (Dworkin et al., 2017; Taylor et al., 2018; Clifton-Brown et al., 2019; Jiang et al., 2019; Palmer et al., 2019a)]. Switchgrass occurs as upland and lowland ecotypes (Vogel et al., 2011), and some tetraploid switchgrass ecotypes differ in their susceptibility to aphids (Koch et al., 2014a). A lowland switchgrass cultivar Kanlow was resistant to greenbug (*Schizaphis graminum* Rondani., GB) and moderately resistant to the yellow sugarcane aphid (*Sipha flava* Forbes., YSA), whereas an upland switchgrass cultivar Summer was tolerant/moderately susceptible to GB and susceptible to the YSA.

Previous analyses have elucidated global transcriptomic changes occurring in GB infested Summer plants (Donze-Reiner et al., 2017). Principally, GB infestation resulted in an upregulation of genes associated with cell wall receptors, calcium, and reactive oxygen species (ROS) signaling cascades within 5 days after infestation (5DAI), followed by significant upregulation of defense-responsive genes by 10DAI and concomitant downregulation of genes encoding proteins required for primary metabolism. These changes were accompanied by significant increases in hydrogen peroxide levels and activities of peroxidases and laccases. Transcriptional signatures suggested a recovery in plant metabolism by 15DAI, consistent with the tolerant response observed for this plant x aphid interaction (Koch et al., 2014a). Transcriptional evidence implicated jasmonate, salicylate, and ethylene as important mediators of the defense response, similar to what has been reported in other systems (Thaler et al., 2012; Nguyen et al., 2016; Zust and Agrawal, 2016).

In contrast to Kanlow and Summer, a stabilized population of plants derived from random crosses of Summer (♀) x Kanlow (♂) plants (Martinez-Reyna and Vogel, 2008); hereafter referred to as SxK) was susceptible to GB (unlike Kanlow plants) and appeared to be tolerant to YSA (unlike Summer plants) (Koch et al., 2014a). These data suggested that SxK plants individually infested with either GB or YSA could provide important new data on the differential defense responses of hybrid switchgrass.

Here, RNA-seq, gene co-expression, plant hormone, and metabolite analyses were used to elucidate the shared and unique immune networks responsible for hybrid switchgrass defense responses to GB and YSA herbivory.

## Materials and Methods

### Plant Materials

Seeds of an experimental strain, SxK (HP1 C1 High Yield strain) were provided by Dr. Kenneth Vogel, USDA- ARS (Retired), Lincoln, NE.

### Insect Colonies

Colonies for GB (biotype I) and YSA were obtained from Dr. John D. Burd, USDA-ARS (retired) in Stillwater, Oklahoma and maintained on BCK60 sorghum plants as described earlier (Koch et al., 2014b).

### Experimental Conditions and Sample Collection

SxK plants were grown from seed in a greenhouse as previously described (Donze-Reiner et al., 2017; Koch et al., 2018). The plants were arranged in a 3 x 3 factorial design consisting of three treatments (GB-, YSA-infested, and control) and three harvest time points, 5-, 10-, and 15-days after infestation (DAI) and consisted of three biological replicates, with each replication containing four individual plants per treatment per time point. Ten apterous GB or YSA were placed on their respective plants at the onset of the experiment (day 0). To confine aphids, both infested and control plants were caged individually with tubular plastic cages (4 cm diameter by 46 cm height) with vents covered with organdy fabric. Insect damage ratings were performed a minimum of four times with at least three replicates per treatment per timepoint. Tissue was collected from one experimental set of samples with three biological replicates per treatment and per time point. Each biological replicate consisted of four individual genotypes (plants) pooled together. Collected tissues were subsequently processed for transcriptomic, plant hormone, metabolomic, and flavonoid analyses.

Prior to harvesting leaf samples at each time point, aphids were removed with sterile fine-tipped paint brushes and counted. Injury to plants resulting from aphid infestation was assessed using a visual damage rating based on a 1–5 scale *(* Heng-Moss et al., 2002; Koch et al., 2014b). At harvest, all leaves present on plants were collected, flash frozen with liquid nitrogen and stored at -80°C until processed.

### RNA-Seq and Bioinformatics

Total RNA was isolated, purified, and quantitated from flash frozen plant tissue samples and sequenced as previously described (Palmer et al., 2015; Donze-Reiner et al., 2017). Single end 100-bp reads were mapped to version 4.1 of the switchgrass genome (phytozome.jgi.doe.gov) (Goodstein et al., 2012) using HISAT2 (Kim et al., 2015), and read counts were generated using featureCounts (Liao et al., 2014). Differential expression analysis was performed using DESeq2 (Anders and Huber, 2010; Love et al., 2014) in R (Team, 2011). Pairwise contrasts between control and aphid infested plants at each time point were used to generate a list of differentially expressed genes (DEGs) for the entire dataset using an FDR of <0.05 and a fold change of >2 as significance thresholds. The variance stabilization transformation function in DESeq2 was used to generate normalized expression counts for use in subsequent network and heatmap analyses. Co-expression analysis was done by generating signed networks using the weighted gene co-expression network analysis (WGCNA) package (Langfelder and Horvath, 2008) in R.

TFs that could potentially have a major role in hybrid switchgrass defense responses were identified based on three criteria: (1) TFs were present in the top 10% of gene module membership within each module, (2) TFs were present in the 75^th^ percentile based on peak expression of all expressed genes in the network, and (3) TFs had maximal expression differences between control and infested treatments associated with each module profile, with the assumption that these statistically validated and strongly upregulated TFs would be related to plant defense response. These TFs are hereafter referred to a target TFs. Subsequently, subnetworks of genes that had expression profile similarities with each target TF were defined as genes that were represented within the top 1% of topological overlap matrix (TOM) scores for each TF by WGCNA. Members of each subnetwork were categorized for their predicted protein function and classified into Kyoto Encyclopedia of Genes and Genomes (KEGG) pathways *(* Kanehisa et al., 2014).

### Plant Hormone and Metabolite Analyses

JA, JA-Ile, OPDA, SA, and ABA were extracted from 50 mg of ground tissue in methanol/acetonitrile (1:1 v/v) and analyzed by LC-MS/MS (Pan et al., 2008; Schmitz et al., 2015). Targeted analysis of polar metabolites extracted from 50 mg of ground tissue in 80% methanol was performed by multiple reaction monitoring (LC-MRM-MS) analysis (Yuan et al., 2012; Boone et al., 2017). Metabolites were assigned to pathways using the KEGG (Kanehisa et al., 2016). Flavonoid analyses were performed by the Metabolomics Center at the University of Missouri-Columbia.

### Statistical Analysis

JMP (version 12.2.0, SAS Institute 2015) was used for the statistical analyses of all aphid count, plant damage, plant hormone, and plant metabolite data. One-way ANOVA analysis was used to identify significance (*P*-value < 0.05), followed by Tukey HSD (*P* ≤ 0.05) post-hoc test for mean separation where appropriate. KEGG pathway enrichment within co-expression modules were calculated using the Fisher’s exact test (*P* ≤ 0.05) in R relative to all genes in the network.

### Accession Numbers

Bioproject: PRJNA528943. Run accessions: SRR8792755 - SRHR8792789

## Results

### SxK Plants Were Colonized and Damaged by Both Aphids

**Figure 1:**
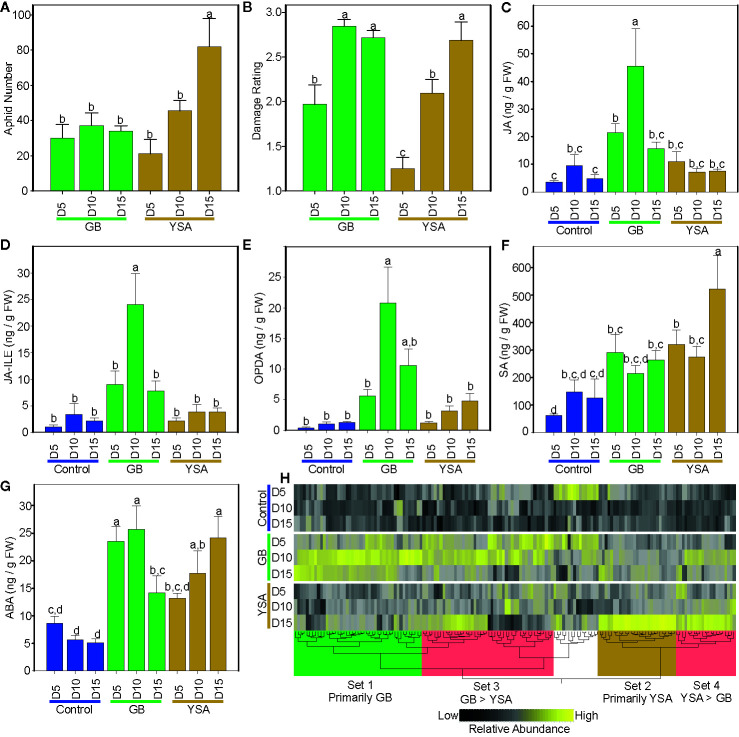
Changes in aphid numbers, plant damage and metabolites across three sampling dates. **(A)** Aphid numbers. **(B)** plant damage ratings. **(C)** Jasmonic acid (JA). **(D)** JA-isoleucine (JA-Ile). **(E)**12-Oxo-phytodienoic acid (OPDA). **(F)** Salicylic acid (SA). **(G)** Abscisic acid (ABA). **(H)** Metabolite heat map, black = low abundance, yellow = high abundance. In **(A)** to **(G)**, green bars are from plants infested with greenbugs (GB), and gold bars are from plants infested with infested with yellow sugarcane aphids (YSA). In **(C)** to **(G)**, blue bars are control uninfested plants. Different letters above bars in **(A)** to **(G)** denote significant differences at *P* ≤ 0.05, with separation of means using Fisher’s LSD. In all panels, days after initial infestation are shown as D5, D10, and D15. In **(H)**, metabolites preferentially enriched in plants infested with GB (set 1, green box), in plants infested with YSA (set 2, gold box), and metabolites enriched in common in infested plants (sets 3 and 4, red boxes). Metabolite lists are provided in **Data S1**.

GB numbers were not significantly different between harvest dates (**Figure 1A**). In contrast, YSA numbers increased significantly with time and were greatest 15DAI (**Figure 1A**). Plants infested with GB were damaged by 5DAI and damage ratings increased to 2.8 ± 0.1 by 10DAI and remained essentially unchanged at 15DAI. For YSA infested plants, damage ratings did not differ from control plants at 5DAI (1.3 ± 0.1) but increased significantly to 2.7 ± 0.2, by 15DAI (**Figure 1B**).

### Aphids Differentially Affect Phytohormones and Metabolite Accumulation

Levels of select plant hormones linked to defense (Nguyen et al., 2016) were quantified by LCMS. JA levels were significantly increased by 5DAI in GB infested plants as compared to control plants and reached maximal values of 45.5 ng g^-1^ fresh weight^-1^ 10DAI before decreasing to levels indistinguishable from uninfested plants (controls) at 15DAI (**Figure 1C**). Levels of the active form of JA conjugated to isoleucine (JA-Ile) were also significantly higher in GB infested plants by 10DAI and decreased in tandem with JA levels by 15DAI (**Figure 1D**). In contrast, JA and JA-Ile levels remained low and did not change under YSA infestation (**Figures 1C, D**). Likewise, OPDA, an intermediate in JA biosynthesis and a signaling molecule involved in plant defense (Heitz et al., 2016 *;* Varsani et al., 2019), was also differentially affected by the two aphids. OPDA trended to be high at all three harvest dates in plants infested with GB with a significant difference observed at 10 DAI as compared to controls. OPDA levels declined by ~50% by 15DAI (**Figure 1E**). OPDA levels were not significantly increased in plants infested with YSAs (**Figure 1E**).

SA levels remained unchanged in GB infested plants with the exception at 5DAI where levels were higher than in controls. In comparison, SA levels in YSA infested plants were significantly higher than controls at both 5DAI and 15DAI (**Figure 1F**). ABA levels were significantly elevated in GB infested plants at 5DAI and 10DAI and remained significantly higher even at 15DAI compared to controls. Conversely, ABA accumulated more gradually in YSA infested plants, with ABA levels significantly different from controls at 10 and 15DAI (**Figure 1G**).

Levels of 155 individual metabolites detected by LCMS were impacted as a result of time or infestation with four major sets of metabolites whose levels were strongly correlated with aphid infestation (**Figure 1H** and **Data S1**). Overall, metabolite levels were consistent with plant damage ratings and peaked at 10DAI and 15DAI for GB and YSA, respectively. Set 1 consisted of metabolites that were primarily elevated in GB infested plants at 10DAI and contained a majority (10 out of 12) of the identified amino acids as well as purines. Set 2 contained several metabolites that were strongly elevated in YSA infested plants at 15DAI and were associated with pathways related to primary carbon metabolism, including glycolysis, the TCA cycle, the pentose phosphate pathway (PPP), and starch and sucrose metabolism (**Data S1**). Sets 3 and 4 consisted of metabolites that were elevated in plants infested by both aphids; however, levels of metabolites in set 3 were generally higher in GB infested plants while metabolite levels in set 4 were higher in YSA infested plants.

### Network Analysis Identifies Key Defense Related Modules of Co-Expressed Genes

Discriminant analysis of transcriptomes indicated developmental-related and aphid-induced changes over the time course of the experiment (**Figure S1A**), and 16,192 DEGs (FDR ≤ 0.05 and a fold change of ≥ 2) as a result of GB or YSA infestation were identified. Hierarchical clustering (**Figure S1B**) revealed gene expression profiles strongly associated with aphid herbivory reinforcing findings detected by global metabolite analyses (**Figure 1H**).

**Figure 2:**
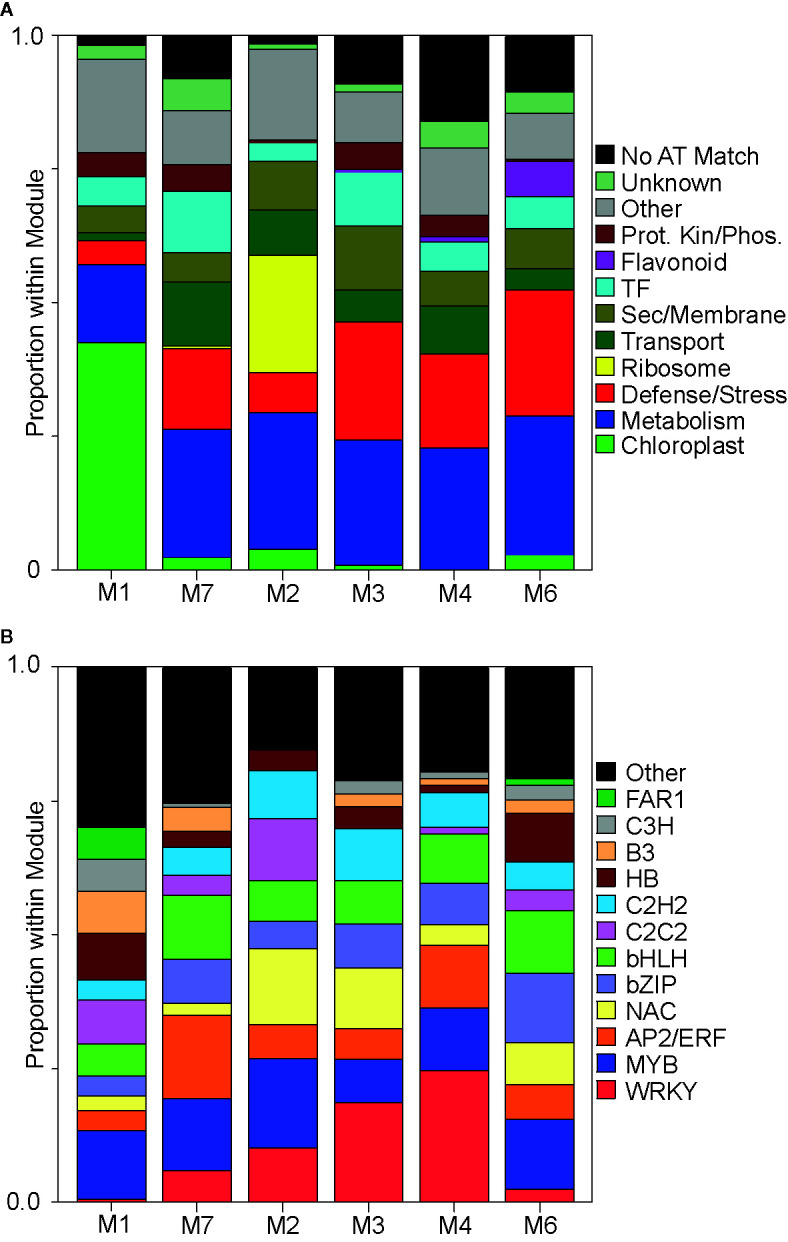
Functional classification of genes within each co-expression module. **(A)** Proportional distribution of the ~top 200 genes within each module. Proteins encoded by these ~top 200 genes were manually assigned into predicted cellular functions. Proteins whose functions were ambiguous were grouped into “other” category, (black color). Assigned functional classifications are in colored boxes to the right of panel (see **Data S1**). **(B)** Transcription factor abundances in the top ~10% of all genes present within each module. Encoded proteins were grouped into known plant transcription factor families [PlantTFDB, http://planttfdb.gao-lab.org/index.php?sp=Pvi (Jin et al., 2017)], which are color coded and shown to the right of panel (also see **Data S1**).

A WGCNA was performed, and six modules of co-expressed genes, hereafter referred to as M1, M7, M2, M3, M4, and M6, were identified whose expression profiles spanned a range of switchgrass interactions with both aphids (**Figure S2**). Module (M) number and co-expression profiles are summarized in **Table 1**. To better define the plausible relationships between the selected gene co-expression modules to switchgrass defense, the top 200 genes (including DEGs and non-DEGs) with the highest gene module membership scores (see Experimental Procedures) within the six co-expression modules were used to interrogate their potential functional roles in plant growth and defense (**Figure 2A** and **Data S1**).

**Table 1:** Relationships of gene co-expression modules and their profiles in aphid infested relative to control uninfested plants.

Module no.	Gene co-expression profile
M1	Upregulated in controls, downregulated by aphids
M7	Upregulated at 5DAI in response to both aphids
M2	Upregulated 5DAI by GB and at 15DAI by YSA
M3	Upregulated by GB all days, not by YSA
M4	Upregulated by GB all days and by YSA at 15DAI
M6	Upregulated by YSA, not by GB

Actual profiles are provided in **Figure S2**.

M1 contained genes upregulated in controls but downregulated by herbivory of both aphids and was enriched in genes related to chloroplast (growth) and depleted for genes associated with defense/stress (**Figure 2A**). M7 contained genes that likely encoded key factors in switchgrass basal defense because they were upregulated maximally at 5DAI in response to both aphids. Genes assigned to this module included orthologs to *Arabidopsis* PSB1, a protein kinase integral to plant defense (Swiderski and Innes, 2001; Sun et al., 2017), calcium channels, several potential calcium sensors, and calcium-dependent kinases (**Data S1**).

M2 contained gene subnetworks likely associated with plant stress responses that were commonly induced by either aphid, occurring earlier under GB infestation (5DAI) and later under YSA infestation (15DAI). A striking feature in M2 was the extensive co-expression of genes associated with ribosomes, and a reduced cohort of genes linked with defense/stress response along with a reduced cohort of genes encoding TFs, components of the flavonoid biosynthesis pathway, and protein kinase/phosphatase as compared to M3, M4, M6, and M7 (**Figure 2A**). M2 was also enriched in genes associated with secretory/membrane processes as compared to M7.

M3 contained genes that were strongly upregulated in response to GB infestation and were largely unresponsive to YSA herbivory and were enriched for genes associated with defense/stress, TFs, protein kinases/phosphatases, and secretory/membrane, such as transporters potentially involved in ABA uptake [annotated as pleiotropic drug resistance 12; **Data S1**; (Kang et al., 2010)], consistent with significantly greater ABA levels at 5DAI and 10 DAI in GB infested plants (**Figure 1G**).

M4 contained subnetworks of genes that were upregulated at all three sampling dates in GB infested plants but were only upregulated at 15DAI in YSA infested plants. Functions of these genes largely mirrored the distributions observed in M3, except M4 contained more genes related to transport and was relatively less populated with genes encoding TFs (**Figure 2A**; **Data S1**). However, like M2, genes annotated as sugar, phosphate, amino acid, and heavy metal ions transporters were also enriched in M4, suggesting some mechanistic commonality in defense responses to the two aphid species.

Genes that were most strongly induced in response to YSA herbivory were found in M6. Predicted functions of proteins assigned to this module were similar to those found in M3; however, unlike M3, M6 contained several genes encoding enzymes involved in flavonoid biosynthesis (**Figure 2A** and **Data S1**). Flavonoid biosynthesis is responsive to SA in other plants (Wozniak et al., 2017), and increased expression of genes associated with flavonoid metabolism could be linked to the significantly elevated levels of SA in YSA infested switchgrass plants, especially at 15DAI (**Figure 1F**).

TF classes downloaded from the Plant Transcription Factor Database, PlantTFDB v5.0 (http://planttfdb.gao-lab.org/index.php?sp=Pvi) were used to identify TFs in the top 10% of the members within each module (**Figure 2A**; **Data S1**). There was a striking underrepresentation of WRKYs and NACs, along with an enrichment of the FAR1, B3, and C3H classes of TFs in M1 (**Figure 2B**). Among TFs classified into “Other” category in M1 was Pavir.4NG231900 encoding a TCP20 homolog. In marked contrast, the five modules associated with plant responses to aphid herbivory were enriched for WRKYs, NACs (except M7), and AP2/ERF classes of TFs.

Enrichment of WRKYs was evident in M7, M2, M3, and M4, all of which contained genes that were upregulated in response to both aphids. In contrast, M6, which contained genes that were largely triggered in response to YSA herbivory, was depleted in WRKYs (**Figure 2B**). Likewise, TFs encoding AP2/ERFs were enriched in the modules associated with response to aphids (**Figure 2B**) with the largest proportions of AP2/ERFs associated with M7 and M4. Both M7 and M4 contained genes whose expression levels were induced by both aphids either at 5DAI (M7) or to GB (all DAIs) and YSA at 15DAI, indicating a link to switchgrass defense responses. MYB TFs were found in all six co-expression modules although their proportions were lower in M3, which contained genes strongly associated with GB herbivory. NACs were also relatively depleted in some of the modules associated with aphid feeding, including M7 and M4 (also M1) but enriched in the other three modules (**Figure 2B**). NACs associated with M2 and M3 could be implicated in response to plant damage induced by aphid herbivory, consistent with plant damage ratings shown in **Figure 1B**. Genes encoding bZIPs and bHLH TFs were likewise enriched in the modules associated with aphid herbivory, with greater enrichment in M7 (early response) and in M6 (associated with YSA herbivory).

Differences in the module proportions of C2C2 and C2H2 classes of TFs were also observed, with greater enrichment of both classes in M2, relative to the other modules (**Figure 2B**). Genes encoding FAR1 TFs were detected only in M6 and M1.

### Subnetwork Analysis Identifies Target TFs Linked to Switchgrass Defense Responses

**Figure 3:**
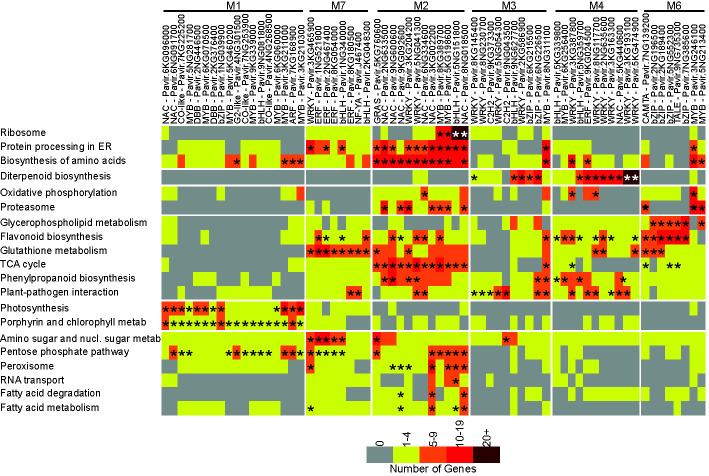
Target TF subnetwork analysis. Modules and target TFs with family and gene id. are shown at the top of the figure. KEGG pathways showing subnetwork enrichment are shown on the left. The number of genes associated with individual TFs in each KEGG pathway are color coded, from gray – no genes to brown – >20 genes. Asterisks denote significant enrichment within the subnetwork at *P* ≤ 0.05.

Target TFs and their co-expressed genes were determined as described in the experimental procedures section. Altogether, genes encoding proteins involved in twenty different KEGG pathways were found to be significantly enriched in one or more target TF subnetwork (**Figure 3**) relative to all genes in the entire network.

Eighteen target TFs were identified in M1 (control plants). Statistically significant enrichments were found for biosynthesis of amino acids with four TFs, photosynthesis with 12 TFs, porphyrin and chlorophyll metabolism with all 18 highly expressed TFs, and pentose phosphate pathway with 12 TFs. Gene expression subnetworks linked two MYBs, Pavir.3KG211000 and Pavir.3KG210300, and one ARF ortholog to multiple metabolic processes, suggesting a direct role for these target TFs in switchgrass growth responses.

The M7 co-expression module, upregulated in response to both aphids at 5DAI, contained eight target TFs whose subnetworks were all associated with glutathione metabolism. Four out of these eight TFs encoded ethylene response factors (ERFs) orthologous to *Arabidopsis* RAP2.12 and 2.6 proteins, and their subnetworks were significantly linked to pentose phosphate pathway and amino sugar and nucleotide sugar metabolism. Subnetworks associated with two other ERFs, Pavir.1NG521800 and Pavir.2NG476400, were enriched in genes associated with flavonoid metabolism (**Figure 3**). Other target TFs in M7 included two bHLH orthologous to *Arabidopsis* ASK2 (AT1G05805) and PIF3 (AT2G03340), a nuclear factor Y subunit A6 homolog whose subnetworks included genes associated with plant-pathogen interactions, and starch and sucrose metabolism. A WRKY-encoding gene strongly induced at 5DAI by GB and YSA feeding was significantly linked to genes encoding proteins involved in protein processing in endoplasmic reticulum, amino sugar and nucleic sugar metabolism, pentose phosphate pathway, and peroxisome (**Figure 3**).

M2 consisted of genes that were upregulated in response to GB at 5DAI and YSA at 15DAI and contained twelve target TFs belonging to the NAC (6), WRKY (2), MYB (2), bHLH (1), and GRAS (1) families (**Figure 3**). Four of the six switchgrass NACs were homologous to *Arabidopsis* ATNAP, a NAC that regulates leaf senescence in *Arabidopsis* (Guo and Gan, 2006). Subnetworks associated with these NACs were also enriched genes related to protein processing in the endoplasmic reticulum, biosynthesis of amino acids, proteasome, TCA cycle, phenylpropanoid biosynthesis, and peroxisome, among others. Subnetworks for the two WRKYs in M2 were enriched in genes involved in biosynthesis of amino acids, proteasome, and the TCA cycle. Notably, the bHLH gene in M2 was significantly associated with genes classified into the ribosome KEGG pathway and several other metabolic processes potentially related to ribosomal function, such as protein processing in the endoplasmic reticulum, biosynthesis of amino acids, and RNA transport, suggesting an important role for this TF in switchgrass defense response.

Predominantly, linkages in M3 occurred between TFs and pathways for diterpenoid biosynthesis and plant-pathogen interaction. In addition, a C2H2 TF encoded by Pavir.9NG043500 was significantly associated with amino sugar and nucleic acid sugar metabolism, Pavir.6NG226500 encoding a bZIP was significantly associated with phenylpropanoid biosynthesis, and one MYB was significantly associated with protein processing in the endoplasmic reticulum, biosynthesis of amino acids, flavonoid biosynthesis, TCA cycle, and phenylpropanoid biosynthesis, but not diterpenoid biosynthesis.

Six WRKYs were among the 11 target TFs in M4. Significant associations for five of these six WRKYs were to diterpenoid biosynthesis with more variable associations to other metabolic processes, including oxidative phosphorylation, phenylpropanoid biosynthesis and plant-pathogen interaction. Similarly, one ERF and one NAC were also associated with multiple processes (**Figure 3**).

The YSA specific M6 contained eight target TFs, with four bZIPs, two MYBs, and one each of CAMTA and TALE TFs (**Figure 3**). The most significant associations between these TFs and metabolic processes were for glycerophospholipid metabolism and flavonoid biosynthesis. Two bZIPs, Pavir.2NG96500 and Pavir.2KG189400, were also significantly linked to glutathione metabolism. The two MYBs in M6 were linked to biosynthesis of amino acids and proteasome. Differences in the target TFs connections in M3 responding to GB herbivory and M6 responding to YSA herbivory suggest distinctive defense responses of hybrid switchgrass to the two different aphids.

### Aphid Herbivory Coordinately Changes Pathways and Metabolite Enrichment

**Figure 4:**
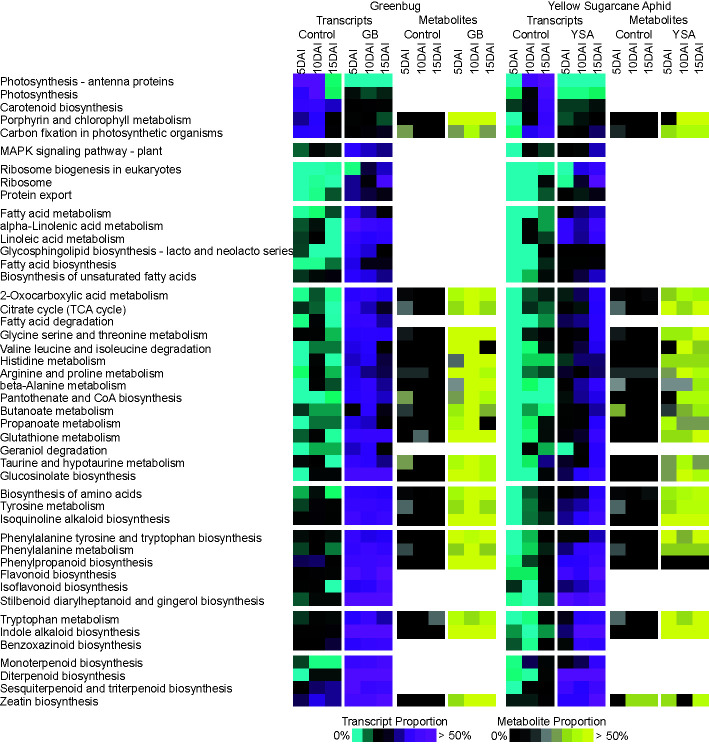
Coordinate changes in transcripts and metabolites associated with KEGG metabolic pathways in response to aphid herbivory. Transcripts were assigned to KEGG pathways based on their provided KO and EC annotations in Phytozome. Relative enrichment of transcripts and metabolites were compared between control uninfested plants to plants infested with greenbugs (GB) or yellow sugarcane aphids (YSA) at the three sampling times. Transcript proportions (number of genes upregulated in control or aphid infested plants relative to all expressed genes in that pathway) in each pathway identified is shown from 0% - cyan to >50% - magenta. Metabolite proportion (number of metabolites more abundant in control or aphid infested plants relative to all detected metabolites in that pathway) is shown from 0% - black to >50% yellow.

Pairwise comparisons of DEGs encoding proteins associated with KEGG pathways and metabolites enriched within each pathway was next performed to identify pathways that were most significantly associated with aphid infested and control plants (**Figure 4**). It should be noted that genes and metabolites were not uniquely and exclusively associated with one pathway. Pathway occupancy was defined as a percentage of the number of DEGs or differentially abundant metabolites in pathway/total number of expressed genes or detected metabolites in the KEGG pathway and was used to visualize changes occurring between controls and treatments.

Control plants had an enrichment of pathways related to photosynthesis and photosynthetic pigment biosynthesis, especially at 5DAI and 10DAI, as compared to GB infested plants. However, primary metabolites linked to porphyrin and chlorophyll metabolism (glutamate and threonine) and carbon fixation (sugars) were enriched in GB infested plants, which could have arisen from dissimilatory routes. Pathways linked to defense perception, signaling, and metabolic redirection were enriched in GB infested plants. These included MAPK signaling, ribosome biogenesis, protein export, and fatty acid metabolism. Similarly, several primary and secondary metabolic pathways and associated metabolites were significantly enriched by 5DAI in GB infested plants relative to controls (**Figure 4**). Notably, these included valine, leucine, and isoleucine degradation; arginine and proline metabolism; and tyrosine metabolism, with concomitant increases in levels of associated amino acids and their oxo-acid breakdown products. For tyrosine, tryptophan, and phenylalanine, genes associated with their biosynthetic and degradative pathways as well as their associated metabolites were upregulated by GB herbivory. Additionally, pathways that utilize these three amino acids as precursors for the biosynthesis of secondary defense metabolites, such as those derived from the phenylpropanoid, flavonoid, stilbenoid, indole alkaloid, and isoquinoline alkaloid biosynthetic pathways, were also all strongly upregulated by GB feeding. Terpenoid biosynthetic pathways were similarly upregulated, indicative overall of a strong defense response and diversion of products of primary plant metabolism to the formation of defensive compounds.

Delayed changes to expression of genes associated with primary and secondary metabolism were found under YSA infestation with maximal gene expression levels frequently observed at 15DAI. For example, several pathways including fatty acid metabolism; alpha-linoleic metabolism; fatty acid biosynthesis; and glycine, threonine, and serine metabolism were enriched at 5DAI in GB infested plants and at 15DAI under YSA infestation. Furthermore, as compared to GB-switchgrass interactions, in the YSA-switchgrass interactions, metabolites and transcripts associated with porphyrin and chlorophyll metabolism and carbon fixation did not differ from control plants until 10DAI and 15DAI. These data were consistent with limited plant damage observed at 5DAI in hybrid switchgrass infested with YSA with increase in damage score and aphid numbers recorded until 15DAI. YSA infestation until 15DAI. In many of these instances, metabolite enrichment tracked with pathway enrichment (**Figure 4**).

Other differences were also evident in plant responses to the two different aphids. Histidine levels were strongly elevated in GB infested plants and only moderately elevated in YSA infested plants. Such differences were also seen for metabolites associated with the β-alanine, taurine and hypotaurine, phenylalanine, and phenylpropanoid metabolism (**Figure 4**).

### Other Defense-Related Molecular Signatures of GB and YSA Herbivory

Recently, (Koch et al., 2018) did not observe any correlations between aphid feeding and callose deposition or the expression profiles of specific callose synthase genes after 3DAI. In the current studies performed over a longer duration, expression profiles of these same genes yielded similar results (**Figure S3**).

**Figure 5:**
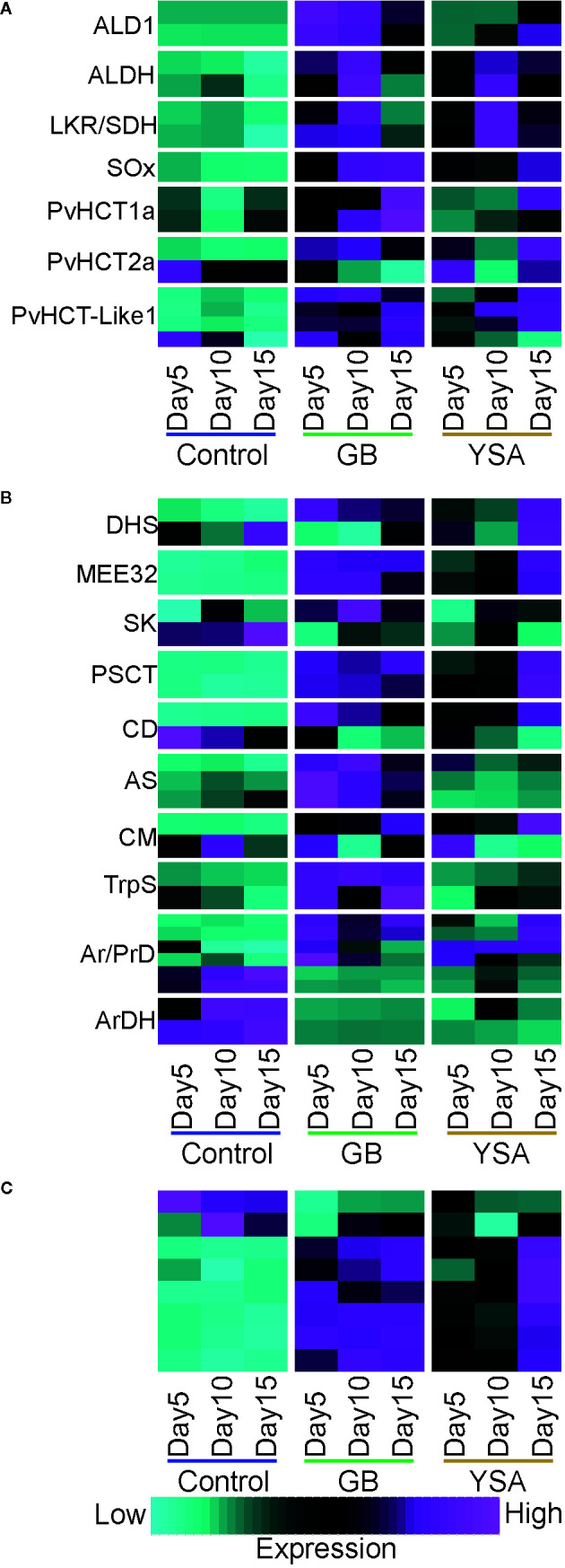
Expression heat maps of genes required for biosynthesis of select defense-related compounds. Maps are based on z-scores of DEGs where cyan is low expression and magenta is high expression. **(A)** Pipecolic acid and caffeoyl conjugate biosynthesis. **(B)** Shikimate and aromatic acid pathways. **(C)** Enolases. Gene abbreviations and locus information are provided in **Data S1**.

Both GB and YSA herbivory significantly elevated levels of pipecolic acid by 5DAI. Once induced, these levels remained high throughout the time-course of the experiment (**Data S1**). Genes encoding enzymes involved in the biosynthesis of pipecolic acid (ALD1, ALDH, LKR/SDH, and SOx; **Figure 5A**), and several genes encoding hydroxycinnamoyl CoA-quinate transferases required for the biosynthesis of chlorogenic acid and related compounds were also significantly upregulated by 5DAI (HCT; **Figure 5A**), corroborating earlier findings (Donze-Reiner et al., 2017).

Several other amino acids, sugars, and metabolites associated with vitamin and nucleic acid metabolism also accumulated in GB and YSA infested plants, supporting an upregulation of genes encoding hydrolases and a concomitant downregulation of genes involved in biosynthesis of primary macromolecules. In most cases, peak expression levels were observed at 10DAI with subsequent decreases by 15DAI in GB infested plants, whereas greatest enrichment was often observed at 15DAI for YSA infested plants (**Data S1**). Methionine sulfoxide, a byproduct of oxidative stress, was significantly enriched by 5DAI in GB infested plants and by 10DAI in YSA infested plants, as was 4-amino butyrate (GABA) at all harvest dates under GB infestation and only at 5DAI under YSA infestation, although levels of GABA increased with time in YSA infested plants (**Data S1**).

Levels of Phenylalanine (Phe), tyrosine (Tyr), and tryptophan (Trp), key amino acid precursors for several plant secondary metabolites, were also changed in response to aphid herbivory (**Data S1**). Levels of Phe and its breakdown product phenyllactic acid were significantly enriched only in plants infested with GB. However, prephenate, another breakdown product arising from Phe and an intermediate for chorismite biosynthesis, was significantly elevated at 15DAI in YSA infested plants, indicating that potential differences in how plant secondary metabolism was affected by these two aphids. Levels of Tyr were significantly different only at 10DAI in hybrid switchgrass plants infested with GB, and no significant changes in Tyr levels were detected in YSA infested plants (**Data S1**).

Evidence for both accumulation and breakdown of Trp were detected. Trp levels were significantly elevated at all sampling dates in plants infested with GB and YSA, with a continued increase in levels observed from 5DAI to 15DAI under YSA pressure, although levels of Trp declined by approximately 37% between 10DAI and 15DAI in GB infested plants (**Data S1**). Genes encoding tryptophan synthases were moderately upregulated under GB herbivory, but their expression levels were relatively unaffected by YSA feeding, and no other obvious differences in expression patterns in other genes associated with this pathway were observed in plants infested with YSA (**Figure 5B**). Products of Trp degradation, kynurenine and anthranilate, were significantly elevated in plants infested with both aphids, with greater levels of anthranilate observed under YSA infestation. Among other pathways, anthranilate is required for quinazoline alkaloid biosynthesis, suggesting these alkaloids as potential switchgrass defensive compounds.

Levels of hydroxyphenylpyruvate, an intermediate for Tyr, Phe, and alkaloid biosynthesis, were significantly upregulated at all time points under aphid herbivory. Genes related to aromatic amino acid metabolism were differentially induced by each aphid (**Figure 5B**). Accumulation of 2-dehydro-D-gluconate and D-gluconate in plants infested with GB and YSA indicated the activation of the PPP (**Data S1**). However, other metabolites linked to PPP were significantly elevated mostly in YSA infested plants relative to plants infested with GB, including 3-phosphoglycerate, 6-phospho-D-glucono-1,5-lactone, and sedoheptulose-1-7-phosphate. Erythrose-4-phosphate levels, a precursor required for shikimate biosynthesis was not significantly different in any treatment, although genes linked to shikimate biosynthesis were frequently upregulated 5DAI under GB infestation and generally at 10- and 15DAI under YSA pressure. Phosphoenolpyruvate, an essential intermediate for the shikimate pathway, was significantly enriched 5DAI under GB infestation and at 5DAI and 15DAI under YSA infestation (**Data S1**). Genes encoding enolases (**Figure 5C**) were enriched by aphid herbivory, suggesting a path to enhanced generation of phosphoenolpyruvate. Altogether, these data supported an enhanced flux of carbon to terpenoid, phenylpropanoid, and flavonoid pathways.

**Figure 6:**
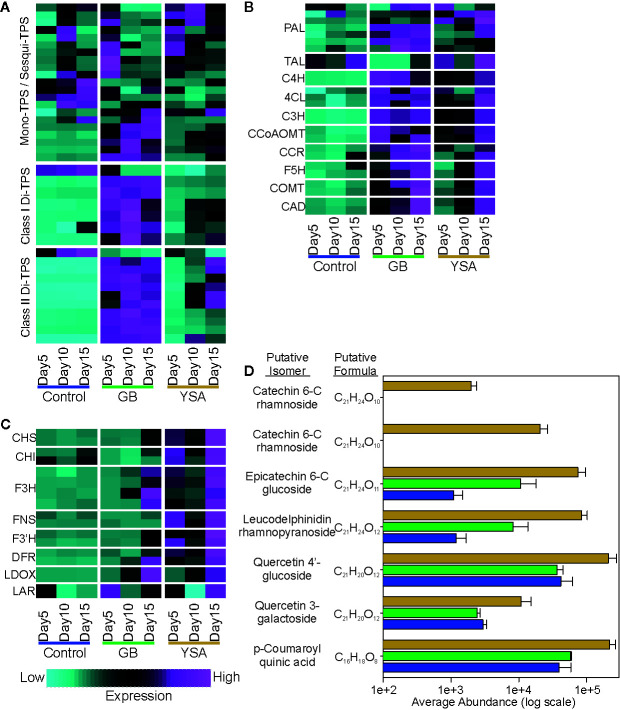
Expression heat maps of select genes associated with biosynthesis of secondary metabolites. Maps are based on z-scores of DEGs where cyan is low expression and magenta is high expression. **(A)** Terpene synthases (TPS). **(B)** Phenylpropanoid pathway genes. **(C)** Flavonoid and anthocyanin pathway genes. **(D)** Abundance of putative flavonoids in plant tissues at 15DAI. Blue bars, control plants; green bars, GB infested plants; gold bars, YSA infested plants. Gene lists are provided in **Data S1**.

Upregulation of genes associated with terpenoid, phenylpropanoid, and flavonoid pathways was detected in hybrid switchgrass plants under insect pressure from both aphids (**Figure 6**). Recently, switchgrass terpene synthases (TPS) have been classified, many have been biochemically characterized, and several shown to be induced after herbivory by fall armyworm (*Spodoptera frugiperda*) (Pelot et al., 2018; Muchlinski et al., 2019). GB and YSA feeding induced upregulation of several TPS genes (**Figure 6A**). There was a significant induction of specific mono/sesqui TPS genes by 5DAI in response to both aphids. Sustained upregulation of class I and class II di-TPS through 10DAI and 15DAI was more prevalent in plants subjected to herbivory by GB as compared to YSA (**Figure 6A**), although a cluster of class II di-TPS genes were upregulated by both GB and YSA herbivory. Similarly, expression of genes encoding enzymes of the phenylpropanoid pathway were upregulated by GB and YSA herbivory. As with the TPS genes, GB herbivory induced a rapid upregulation by 5DAI, whereas maximal expression of these genes was observed at 15DAI in plants infested with YSA (**Figure 6B**). Genes associated with flavonoid biosynthesis were significantly enriched under YSA herbivory (**Figure 6C**), consistent with the apparent diversion of carbon to support flavonoid biosynthesis in these plants. To determine whether upregulation of these genes was associated with altered levels of these metabolites, flavonoids present in plants at 15DAI were analyzed by LCMS (**Figure 6D**). Several putative flavonoids were significantly enriched in plants subjected to YSA herbivory and included isomers of catechin, epicatechin, leucodelphinidin, and quercetin glucosides. p-Coumaroylquinic acid, a product of the condensation of p-coumaroyl-CoA with quinic acid catalyzed by hydroxycinnamoyl CoA-quinate transferases, was also enriched at 15DAI in YSA infested switchgrass plants (**Figure 6D**). A gene encoding a cytochrome p450s annotated as coumaroylquinate(coumaroylshikimate) 3’-monooxygenase (CYP98A3, C3’H; Pavir.3KG235800) that can convert p-coumaroylquinic acid to chlorogenic acid was also significantly upregulated in YSA infested plants, suggesting that both HCT-like and CYP98A3 related enzymes are part of the defense response genes in switchgrass.

## Discussion

Plant responses to aphids can be highly variable based on the genetics of the host and the aphids (Koch et al., 2016; Zust and Agrawal, 2016). GB was unable to utilize the lowland cultivar Kanlow as a host but caused significant damage to the upland cultivar Summer. YSA could colonize both cultivars, although plant damage was always greater on Summer plants (Koch et al., 2014a; Koch et al., 2014b). Here, the potential host networks contributing to the defense response of a hybrid switchgrass, derived from crosses between Summer and Kanlow plants, were evaluated. This stabilized hybrid was colonized and damaged by GB and YSA (Koch et al., 2018). Previously, the defense responses of Summer plants to GB infestation had been studied (Donze-Reiner et al., 2017), but detailed transcriptomic or metabolomic plant responses to YSA herbivory have yet to be reported in the literature.

GB herbivory is generally accompanied by severe host responses *(* Burd and Porter, 2006; Zhang et al., 2019). Virulence associated with GB herbivory has been linked to toxins present in aphid saliva (Nicholson and Puterka, 2014), and damage to switchgrass plants detected by 5DAI was consistent with a virulent response. Conversely, YSA herbivory increased plant damage over the time course of the experiment and likely reflected the sustained loss of plant nutrients required to support increased aphid numbers. GB numbers did not change significantly over the 15 days of the experiment and damage ratings stabilized by 10DAI. A virulent response to YSA has not been reported, although reddish discoloration of leaves, through accumulation of anthocyanins, has been attributed to YSA herbivory of *Sorghum halepense* (L.) Pers. (Costa-Arbulu et al., 2001; Gonzales et al., 2002).

Aphids activate initial plant basal defenses that respond to mechanical and probing stimuli. These responses include calcium fluxes, cell wall perturbation, activation of MAP kinases, detection of aphid-dependent elicitor molecules, downregulation of photosynthesis and assimilation of nutrients, and an upregulation of catabolic processes (Kerchev et al., 2012; Nicholson and Puterka, 2014; Hettenhausen et al., 2015; Vincent et al., 2017; Erb and Reymond, 2019; Xiao et al., 2019). Another common denominator in plant-aphid interactions is the generation and perception of ROS and related signals (Kerchev et al., 2012; Foyer et al., 2015; Koch et al., 2016). Either directly or indirectly these early signals induce changes in plant hormone levels and precipitate longer term defensive changes (Erb et al., 2012). The 5DAI switchgrass responses to both aphids contained molecular signatures indicative of these signaling patterns. Notably, genes encoding wall-associated kinases, MAPKs, RBOHs, and EF-Tu receptor were upregulated (**Figure S4**), and corroborated data obtained with Summer switchgrass x GB interactions (Donze-Reiner et al., 2017). Also, among these early responses were differential changes in hormone levels in response to the two aphid species.

The lack of change in JA, JA-Ile, and OPDA levels in response to YSA but not to GB herbivory was somewhat unexpected, because oxylipins are a critical component of plant responses to wounding and herbivory (Wasternack and Strnad, 2018; Varsani et al., 2019; Yang et al., 2019), although aphids can modulate plant hormone levels to improve feeding outcomes (Thaler et al., 2012; Erb and Reymond, 2019; Yang et al., 2019). There was a stronger induction of genes encoding both 9- and 13-lipoxygenases (LOX) that could contribute to JA biosynthesis in plants infested with GB relative to plants infested with YSA (**Figure S5**). However, in addition to JA and OPDA, oxylipins produced by LOX activities can be converted to other products such as green leaf volatiles (Mochizuki et al., 2016; Varsani et al., 2019; Yang et al., 2019). Whether there was a diversion of oxylipins to other compounds in YSA infested switchgrass is yet to be determined.

ABA and SA are also important hormones impacting plant responses (Hillwig et al., 2016; Kersch-Becker and Thaler, 2019; Yang et al., 2019). ABA was induced early in response to GB and later in response to YSA, while SA was induced solely in response to YSA. Potentially, these changes reflected the ability of YSA to suppress JA-related defenses. Interestingly, weak correlations between expression of genes associated with ABA and SA biosynthesis and levels of these hormones were observed in this study, suggesting that mobilization of stored or transported sources of these hormones could have occurred during aphid herbivory. Both SA and ABA can be mobilized from distal sources, including roots, and/or converted from inactive esters to active forms *via* the action of hydrolases (Seiler et al., 2011; Maruri-Lopez et al., 2019). ABA has also been associated with increased aphid colonization of plants (Studham and MacIntosh, 2013; Chapman et al., 2018), and this appears to be the case in switchgrass as well. In addition, several switchgrass homologs of PDR12 (AT1G15520), which functions as an ABA transporter (Boursiac et al., 2013), were upregulated in plants subjected to aphid herbivory, suggesting mobilization from other tissues and/or stored forms as potential sources of increasing shoot ABA levels. Regardless of the sources of hormones, upregulation of several genes that respond to elevation in ABA, SA, and JA levels, such as those encoding pathogen responsive proteins (PR), plant protease inhibitors, and dehydrins (**Figure S5**), indicated that increased hormone levels indeed influenced plant metabolism.

Despite the differences in hormonal responses to GB and YSA, both aphids seemed to activate basal defenses. Another early metabolic consequence of herbivory by both aphids was the rapid deceleration of nutrient assimilation and acceleration of catabolic processes as evidenced by the significant increases in amino acids, amino acid precursors, and derivatives and downregulation of genes associated with these processes. These findings are consistent with literature reports on aphid herbivory attenuation of plant primary processes, such as photosynthesis, carbon and nitrogen assimilation, and the increase in dissimilatory processes (Wu and Baldwin, 2010; Louis and Shah, 2015; Erb and Reymond, 2019; Nalam et al., 2019). Increased levels of metabolites derived from amino acids such as pipecolic acid, chlorogenic acid, flavonoids, terpenoids, and other secondary compounds strengthen the linkage of repurposing amino acids to the basal defense response in switchgrass.

Transcriptional and metabolomic changes provided a framework for identifying target TFs that potentiated defense responses in switchgrass that were consistent with changes at the transcriptomic and metabolite levels. Due to the early activation of genes in M7 in response to both GB and YSA, we hypothesize that these TFs provide the foundation for longer-term defense responses. *Arabidopsis* homologs to the M7 TFs respond to biotic stress, changes in hormone levels, and cellular ROS. All conditions occurred by 5DAI in hybrid switchgrass under GB and YSA infestation. Notably, these TFs included a 1:1 ortholog of *Arabidopsis* WRKY3/4, which is induced by pathogens and SA (Lai et al., 2008). WRKYs as a class of TFs are well documented for their roles in regulating response to biotic stress and ABA signaling (Park et al., 2006; Oh et al., 2008; van Eck et al., 2010; Rushton et al., 2012; Van Eck et al., 2014). Other TFs were Pavir.2KG048300 encoding a switchgrass PIF4 homolog of AT2G03340 that integrates different environmental cues with hormone signaling in *Arabidopsis* (Choi and Oh, 2016); Pavir.1NG340000 encoding a bHLH protein homologous to *Arabidopsis* AKS2 (AT1G05805) required for ABA-initiated signaling (Takahashi et al., 2017); and four switchgrass homologs of the *Arabidopsis* RAP2.12 gene (AT1G53910) and related ethylene response factor (ERF) genes that respond to changes in cellular ROS and oxygen content among other changes initiated by stress (Liu et al., 2012; Papdi et al., 2015; Yao et al., 2017).

Further attenuation of the basal defense responses involved several overlapping transcriptional events, presumably regulated by the M7 target TFs. These longer-term changes (>5DAI) included balancing metabolism between senescence and growth, a common theme in plants challenged by insect herbivores (Wu and Baldwin, 2010; Zust and Agrawal, 2017; He et al., 2020). Transcriptional changes consistent with these themes were observed in response to both aphids but tended to occur earlier in response to GB and later in response to YSA, and responses that were unique to either aphid. These changes appear to be controlled by a variety of TFs that included many WRKYs, NACs, MYBs, and bHLHs. As examples, four of the NAC TFs in M2 encoded ATNAP homologs [AT1G69490; (Guo and Gan, 2006)]. These four switchgrass NAC genes were also upregulated during the onset of leaf senescence in switchgrass (Palmer et al., 2015; Palmer et al., 2019b), indicating that certain senescence-related KEGG pathways such as ‘peroxisomes’ and ‘fatty acid degradation’ were induced by aphid herbivory, either due to loss of leaf functions or as a means to reallocate nutrients away from aphid-infested leaves to other sinks. More intriguingly, a grass specific WRKY (Pavir.5NG041300), related to *Arabidopsis* WRKY51 (AT5G64810), was strongly induced in M2. This WRKY gene had minimal expression in control plants, suggesting a direct role in switchgrass defense responses to herbivory. ATWRKY51 is regulated *via* Ca^2+^/calmodulin-dependent phosphorylation and required for JA biosynthesis (Yan et al., 2018), providing some correlative evidence for a plausible role for Pavir.5NG041300 in regulating JA/oxylipin biosynthesis in switchgrass or as a response to changes in cell calcium levels. Similarly, four WRKYs assigned to M4 were homologs of *Arabidopsis* WRKY50 (AT5G26170) and ATWRKY70 (AT3G56400). Notably, ATWRKY70 influences both senescence and defense signaling pathways, providing a link between these two processes (Ulker et al., 2007). ATWRKY50 has been linked to repression of JA-inducible defense response (Gao et al., 2011). Target TFs in M4 were also significantly associated with diterpenoid and phenylpropanoid biosynthesis, which generate a variety of metabolites that can stiffen cell walls, act as antifeedants and serve as attractants to parasitoids, and are often upregulated in response to herbivory (Dinh et al., 2013; Tzin et al., 2015; Donze-Reiner et al., 2017; Muchlinski et al., 2019).

The GB-specific co-expression module M3 was enriched for WRKYs, which could indicate the strong defense response initiated by GB herbivory. As an example, Pavir.2KG586900 encodes a WRKY with homology to ATWRKY46 (AT2G46400). ATWRKY46 has been implicated in several functions including basal defense and osmotic stress (Ding et al., 2015), both conditions expected to occur in switchgrass plants under aphid pressure. Several groups have shown the importance of WRKYs in regulating metabolism in response to biotic stress (Voelckel et al., 2004; Li et al., 2008; Oh et al., 2008; Smith et al., 2010; Van Eck et al., 2014). Among the other target TFs in M3, a bZIP homeologous pair (Pavir.6KG215500 and Pavir.6NG226500) homologous to AT4G34590 (ATGBF6) were associated with genes encoding enzymes involved in diterpenoid metabolism among others, potentially linking these bZIPs to defense networks that spiked in response to GB herbivory. A target MYB TF in M3 encoded by Pavir.8NG311100 is a homolog of ATMYB112 (AT1G48000), which positively regulates anthocyanin biosynthesis and suppresses flavonoid biosynthesis in *Arabidopsis* (Lotkowska et al., 2015). A finding that was consistent with the lack of flavonoid enrichment in switchgrass plants infested with GB. Notably, some genes annotated as encoding leucoanthocyanidin dioxygenases (LDOX) were strongly induced by GB herbivory and shared an expression profile with Pavir.8NG311100. LDOX catalyzes the conversion of leucoanthocyanidins to their O+ forms, which are starting substrates for further anthocyanin biosynthesis (KEGG Flavonoid/Anthocyanin pathways). The co-expression association of Pavir.8NG311100 and LDOX genes suggests that regulation of anthocyanin biosynthesis in switchgrass could be like those documented in *Arabidopsis*.

Genes that were upregulated largely in response to YSA were associated significantly with flavonoid biosynthesis based on KEGG pathway analysis, and those encoding proteins linked to Pi stress. Flavonoids were significantly enriched in YSA infested plants at 15DAI, confirming a correspondence to gene expression. M6 target TFs, including bZIPs, CAMTA, and TALE, which were significantly associated with flavonoid metabolism. Since ABA content was elevated in plants challenged with YSA, these data could reveal new aspects of switchgrass defense. Recently, the intersection of ABA, flavonoid biosynthesis, and bZIP TFs has been proposed to be an important trait of land plants (Brunetti et al., 2019). Pi-starvation response in plants is well characterized (Puga et al., 2017), and several switchgrass genes annotated as “phosphate starvation-induced,” “SPX-domains 1 and 3,” “phosphate transporter,” and “purple acid phosphatase” were significantly induced and part of M6. These data suggest that both flavonoid biosynthesis and Pi-stress response are part of the broader adaptations of defense in switchgrass.

**Figure 7:**
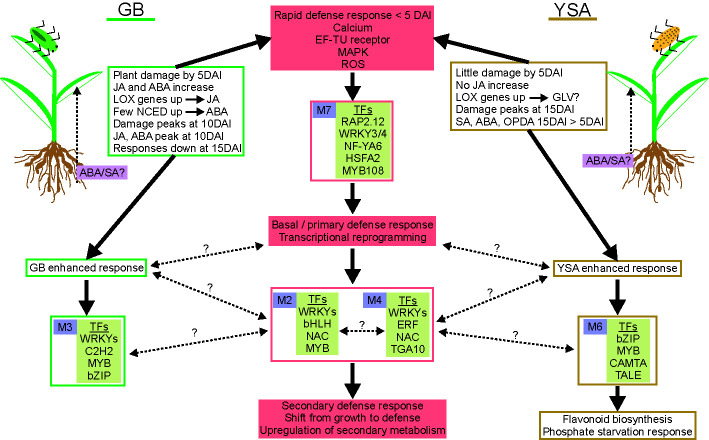
Proposed tentative model integrating primary and secondary defense responses of hybrid switchgrass to GB and YSA. GB is on left and YSA is on right side of the figure. Solid pink boxes show significant aspects of defense responses. Green and gold boxes highlight summary of changes associated with GB (green) or YSA (gold) herbivory. Gene co-expression modules (purple boxes with module number) and significant TFs within each module are identified. GB and YSA-enhanced responses are shown in green or gold boxes, respectively. Solid arrows denote processes for which experimental evidence is presented, and broken black lines with question marks indicate knowledge gaps.

Using a combination of biochemical analysis and bioinformatic approaches, gene co-expression modules, plant hormones, and metabolites that appear to underpin hybrid switchgrass defense responses to aphid herbivory were differentiated. These data are summarized in **Figure 7**. We suggest that these target TFs identified in M7 are part of the basal/primary defense response of switchgrass that subsequently regulate secondary defense responses through other downstream co-regulated and co-expressed gene and metabolic clusters. In addition, our data provided evidence that there were aphid-specific responses (green and gold rectangles, solid arrows, **Figure 7**). Future studies using other pathogens and/or pests could assist in delineating defense pathways that occur in common to biotic stressors and discover exploitable genetic differences in switchgrass plants that could be useful to breeding programs.

## Data Availability Statement

The transcriptomics dataset is available as Bioproject: PRJNA528943. Run Accessions: SRR8792755 - SRR8792789, and is available at the following link: https://dataview.ncbi.nlm.nih.gov/object/PRJNA528943?reviewer=aaqjbu5dkui967krspuuemivdb.

## Author Contributions

TD-R, JB, PT, TH-M, and GS designed the research. KK, TD-R, NP, JS, PT, and GS performed the experiments. KK, NP, EDS, JS, JL, KA, JB, TH-M, and GS analyzed the data. GS, TH-M, JB, KA, and PT provided resources and funding. KK, NP, and GS wrote the manuscript with contributions from all the authors.

## Funding

This work was supported in part by grants from USDA-NIFA grant number 2011-67009-30096 and by the USDA-ARS CRIS project 3042-21000-034-00D. The University of Nebraska DNA Sequencing Core receives partial support from the NCRR (1S10RR027754-01, 5P20RR016469, RR018788-08) and the National Institute for General Medical Science (NIGMS) (8P20GM103427, GM103471-09). This publication’s contents are the sole responsibility of the authors and do not necessarily represent the official views of the NIH or NIGMS.

## Conflict of Interest

The authors declare that the research was conducted in the absence of any commercial or financial relationships that could be construed as a potential conflict of interest.
