# Supplementary figures and images for: Aphid-Responsive Defense Networks in Hybrid Switchgrass
Source: Front Plant Sci. 2020 Jul 30;11:1145. doi: 10.3389/fpls.2020.01145 (PMC7412557; doi:10.3389/fpls.2020.01145)

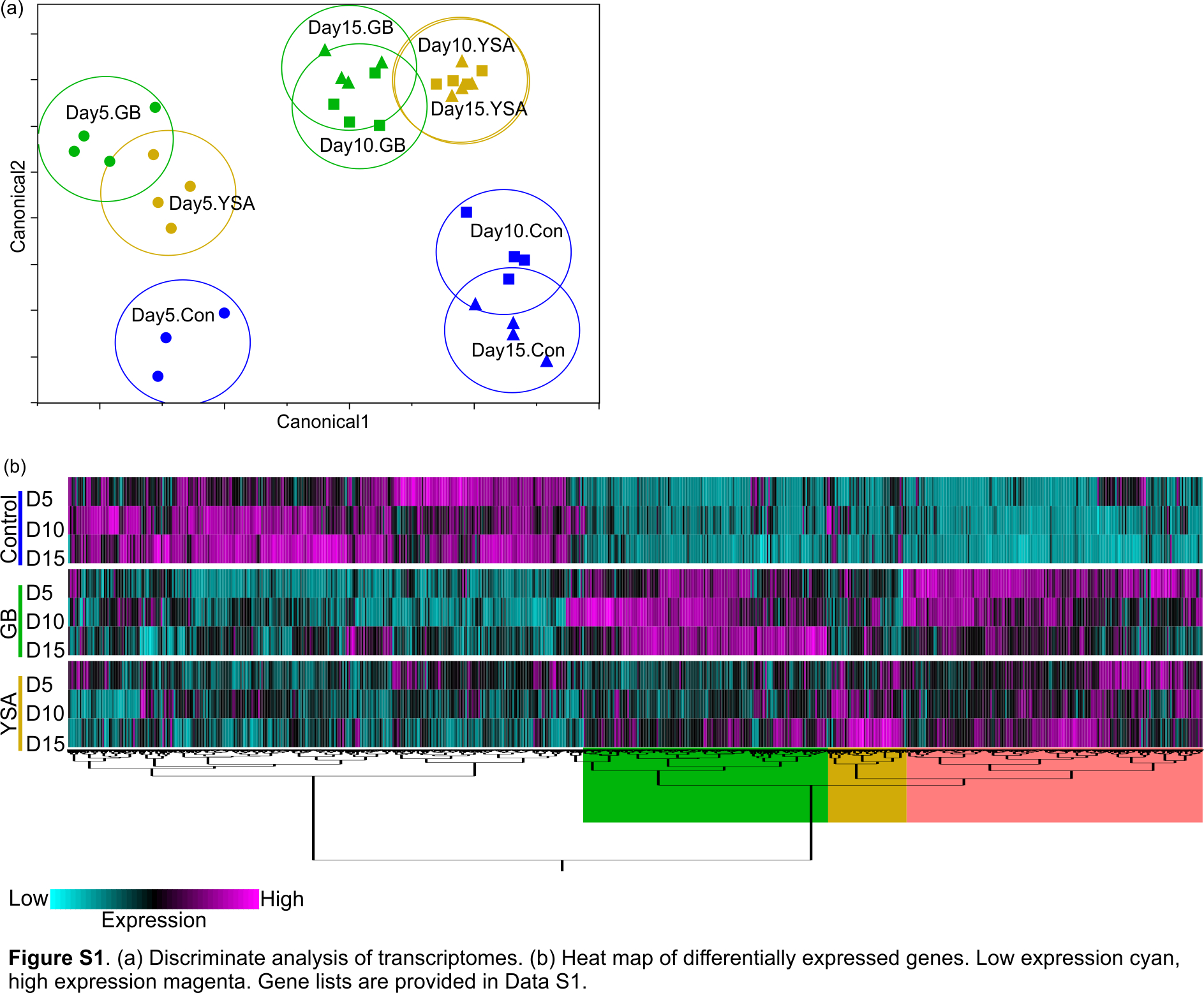

Supplement: Supplementary file 2 [file Image_1.jpeg]

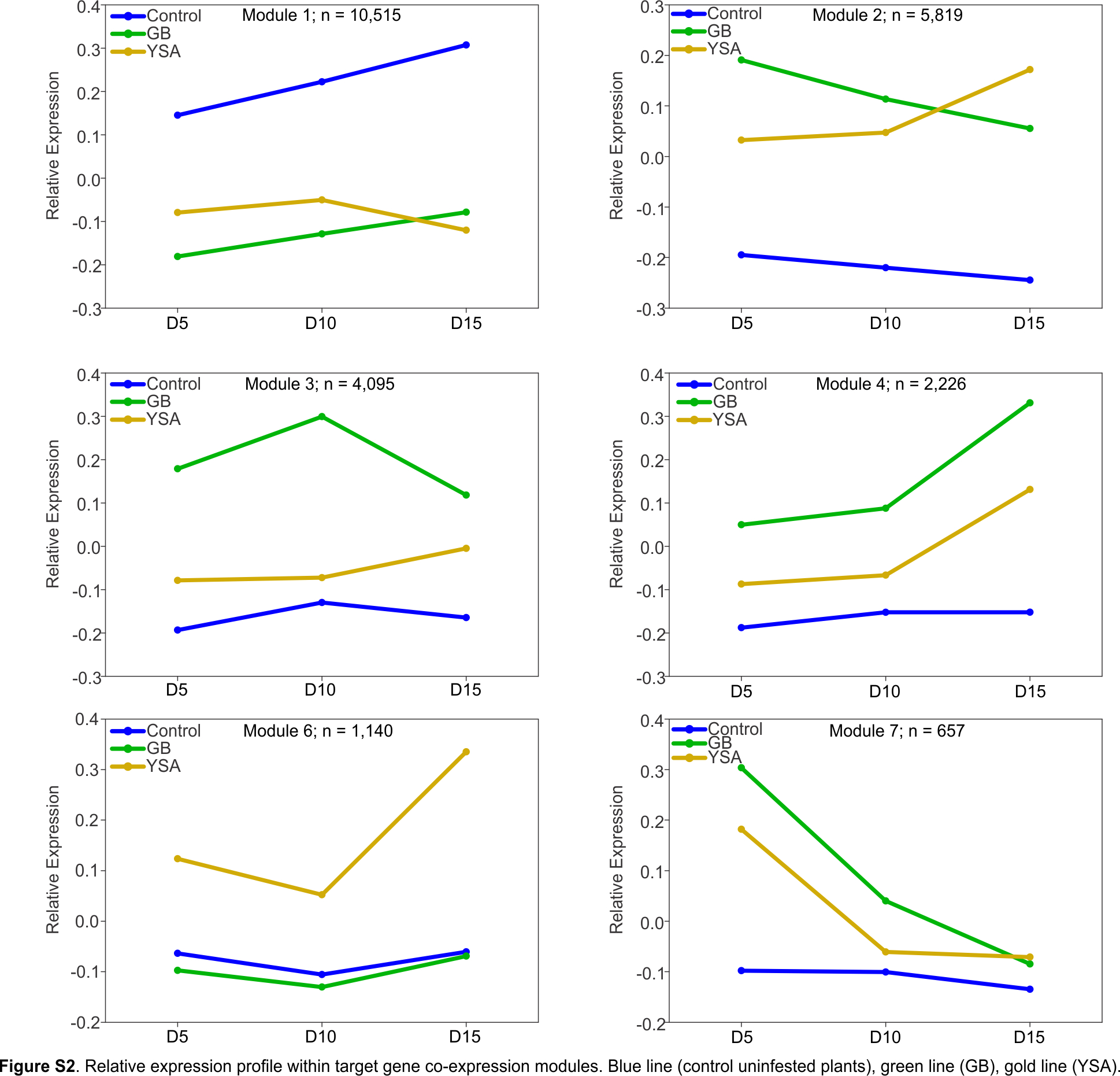

Supplement: Supplementary file 3 [file Image_2.jpeg]

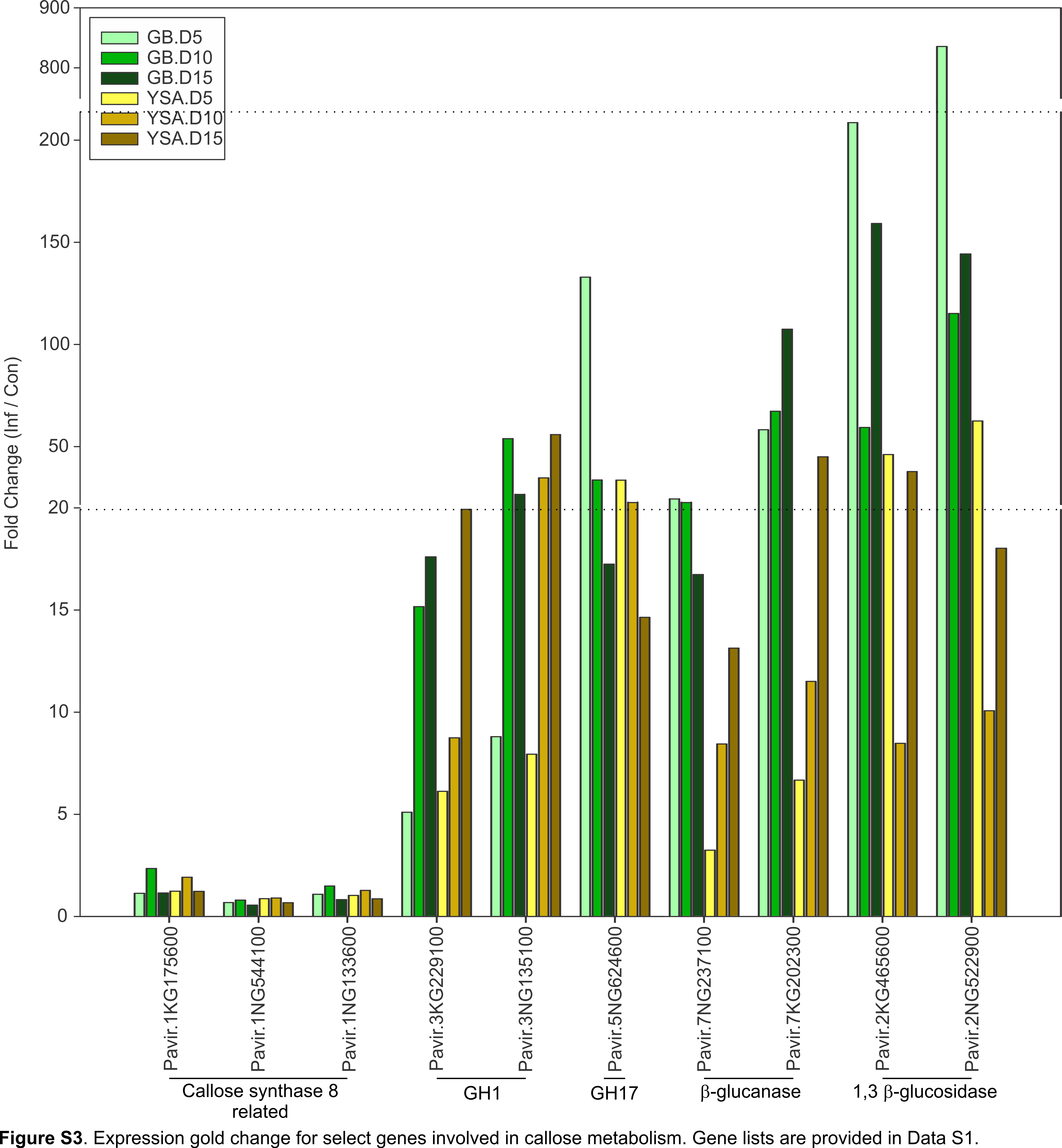

Supplement: Supplementary file 4 [file Image_3.jpeg]

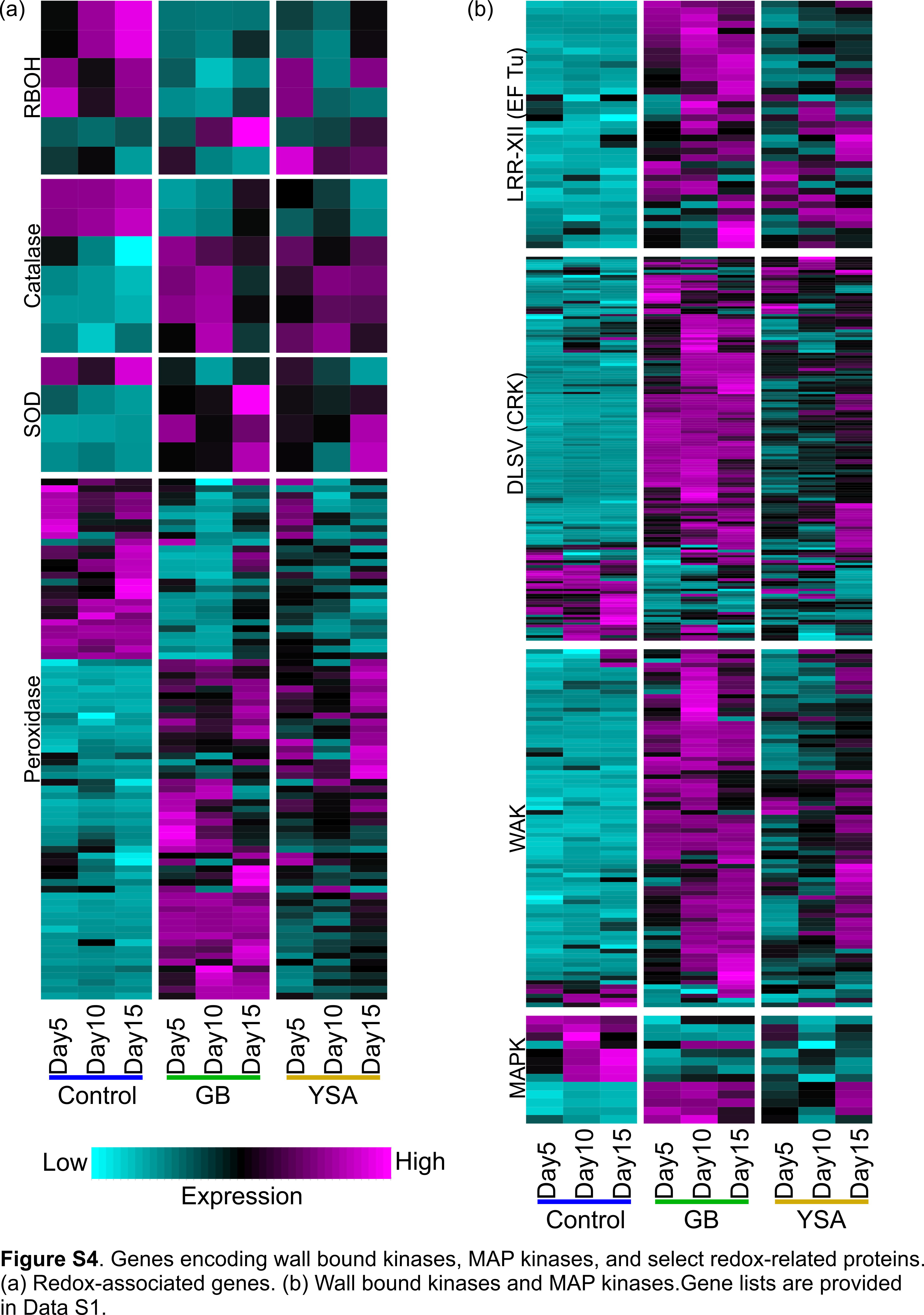

Supplement: Supplementary file 5 [file Image_4.jpeg]

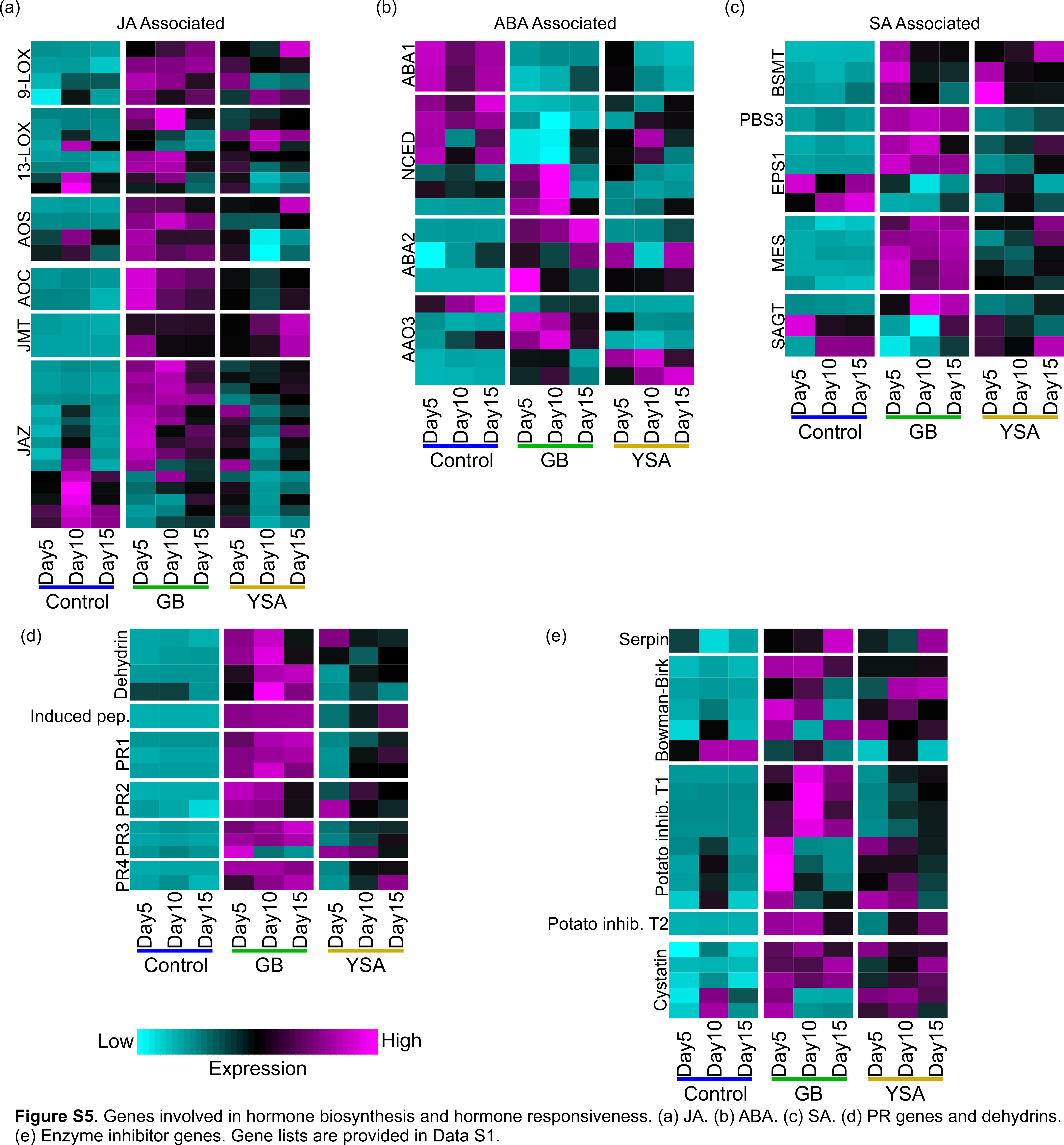

Supplement: Supplementary file 6 [file Image_5.jpeg]
